# A Biochemomechanical Model of Collagen Turnover in Arterial Adaptations to Hemodynamic Loading

**DOI:** 10.21203/rs.3.rs-2535591/v1

**Published:** 2023-02-06

**Authors:** Hailu G. Tilahun, Haritha N. Mullagura, Jay D. Humphrey, Seungik Baek

**Affiliations:** 1 Department of Mechanical Engineering, Michigan State University, East Lansing, MI, USA; 2 Department of Biomedical Engineering, Yale University, New Haven, CT, USA

**Keywords:** growth and remodeling, blood flow regulation, constrained mixture models, collagen turnover

## Abstract

The production, removal, and remodeling of fibrillar collagen is fundamental to arterial homeostasis, including dynamic morphological and microstructural changes that occur in response to sustained changes in blood flow and pressure under physiological conditions. These dynamic processes involve complex, coupled biological, chemical, and mechanical mechanisms that are not completely understood. Nevertheless, recent simulations using constrained mixture models with phenomenologically motivated constitutive relations have demonstrated a capability to predict salient features of the progression of certain vascular adaptations and disease processes. Collagen turnover is modeled, in part, via stress-dependent changes in collagen half-life, typically taken within the range of 10 – 70 days. By contrast, in this work we introduce a biochemomechanical approach to model the cellular synthesis of procollagen as well as its transition from an intermediate state of assembled microfibrils to mature cross-linked fibers, with mechano-regulated removal. The resulting model can simulate temporal changes in geometry, composition, and stress during early vascular adaptation (weeks to months) for modest changes in blood flow or pressure. It is shown that these simulations capture salient features from data presented in the literature from different animal models.

## 1. Introduction

Blood vessels exhibit a remarkable ability to adapt to changes in chemo-mechanical stimuli through evolving vasoactivity and matrix turnover, which can change the luminal diameter, wall thickness, axial stretch, and key mechanical metrics like wall stress and stiffness. The two primary structural constituents within blood vessels are elastic fibers and fibrillar collagens. Elastin endows the wall with an ability to store elastic energy during systole, which can be used to work on the blood during diastole and augment blood flow. Functional elastin is produced primarily before adulthood and under normal conditions has an extremely long half-life, on the order of 50 years. Hence, the normally slow turnover rate of elastin, less than 1 % per year (Wagenseil & Mecham, 2009), is typically negligible. By contrast, fibrillar collagens endow the arterial wall with both stiffness and strength. These fibers are synthesized and degraded continuously, with a normal half-life on the order of 70 days. Their turnover thus contributes both to maintaining arterial integrity over long periods and facilitating adaptations to altered hemodynamic loads. The specific turnover rate can vary with disease and injury, indeed even with changes in mechanical load. Differences between the rates of synthesis and degradation of collagen can lead to fibrosis (as in aging or hypertension) or weakening of the wall (as in dissection or rupture). There is a pressing need, therefore, to capture possible imbalances in collagen turnover over time (Bishop et al., 1994; Bruno et al., 1998; Chyatte & Lewis, 1997; Majamaa & Myllyla, 1993; Rodriguez-Feo et al., 2005; Sluijter et al., 2004a).

Over the past two decades, constrained mixture models of arterial homeostasis and pathogenesis have proven capable of describing and predicting salient features of the progression of diverse vascular responses and conditions (Baek et al., 2007; Braeu et al., 2017; Humphrey, 2021; Wagenseil, 2011). Briefly, these models assume that blood vessels are solid-bounded mixtures wherein individual constituents are incorporated within extant matrix under a pre-stretch and subject thereafter to vasoactive changes and stress-mediated growth and remodeling (G&R). Although these stress-mediated models can capture arterial adaptations and maladaptations over periods of weeks to months or years, the underlying constitutive relations are yet phenomenological (Latorre & Humphrey, 2020; Valentín et al., 2009). By contrast, this paper seeks to extend a previous constrained mixture model of short-term vascular adaptations by incorporating knowledge of the intracellular and extracellular biochemical processes of collagen production and removal.

## 2. Brief Review of Flow- and Pressure-induced Adaptations

### 2.1. Mechanosensitive behaviors

Changes in blood flow and/or pressure under physiological conditions can induce modest alterations in arterial stress (e.g., luminal wall shear stress and intramural axial and circumferential stress), which in turn can change the gene expression profile of the vascular cells (e.g., endothelial, smooth muscle, and fibroblasts) and thereby lead to changes in smooth muscle tone and, if sustained, turnover of extracellular matrix (ECM). Such changes allow the vessel to adapt, which is often homeostatic if the perturbations are modest (Humphrey, 2008). For a normal artery in humans, wall shear stress and circumferential stress tend to be maintained at certain levels, ~1.5 Pa for the former (Ando & Yamamoto, 2013) and ~150 kPa for the latter (Fridez et al., 2003). Stress analyses that include residual stress, basal smooth muscle tone, and nonlinear wall properties suggest that the distribution of axial and circumferential wall stress tend to be nearly uniform across the wall in normalcy (Humphrey, 2002). The mean values of stress are given by:

(1) $$\sigma_{1}=\frac{f_{1}}{\pi h\left(2r_{i}+h\right)},\;\sigma_{2}=\frac{Pr_{i}}{h},\;\tau_{w}=\frac{4\mu Q}{\pi r_{i}^{3}},$$

where *f*_1_, *P*, and *Q* are the axial force, transmural pressure, and volumetric flow rate; *r*_*i*_ and ℎ are the inner radius and thickness of the artery; *μ* is the viscosity of the blood; and *σ*_1_, *σ*_2_ are the axial and circumferential stresses, respectively, and *τ*_*w*_ is the wall shear stress.

Flow-induced wall shear stress is an important mechano-stimulus that regulates the production of vasoactive molecules by the endothelial cells, which in turn control the vasoactive tone of the smooth muscle cells. Increases in flow tend to upregulate endothelial-derived nitric oxide synthase, which catalyzes the synthesis and secretion of the potent vasodilator nitric oxide (NO), which causes smooth muscle cell relaxation (Hsiai, 2008; Lu & Kassab, 2011; Moncada et al., 1989). Conversely, decreases in flow tend to upregulate endothelial synthesis and secretion of endothelin-1 (ET-1), a potent vasoconstrictor that causes smooth muscle cell contraction (Kuchan & Frangos, 1993; Morawietz et al., 2000). Both of these changes determine the state in which ECM turns over.

### 2.2. Arterial collagen synthesis and degradation

Collagen fibrils and then fibers are formed via complex biosynthetic pathways involving intracellular regulation and synthesis and extracellular post-translational modifications, each with respective time scales (Berg et al., 1980; Trackman, 2005). Importantly, smooth muscle cell production of collagen tends to be attenuated by NO but augmented by ET-1 (Humphrey, 2008). In this way, endothelial sensing of sustained changes in flow leads not only to a change in lumen but also a change in collagen turnover within the altered mechanical state (Rudic et al., 1998). If the altered flow is sustained over a long period, G&R can occur in a dilated or constricted configuration and thereby help adapt the wall to the new mechanical stimulus.

**Figure 1.**
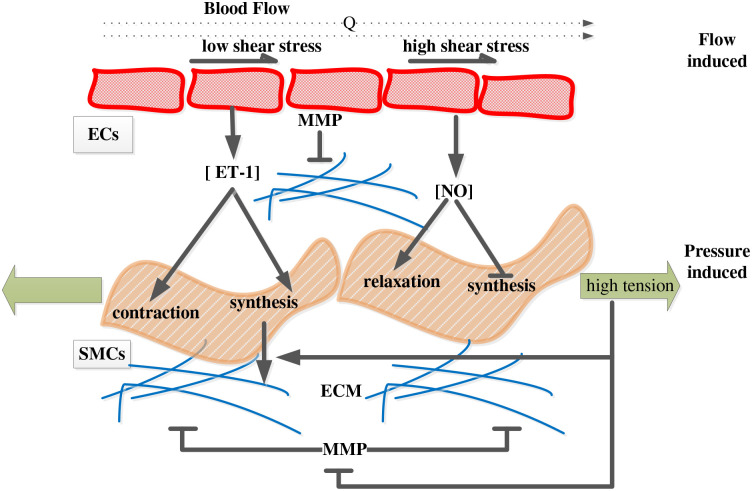
Schematic illustration of effects of hemodynamics on the regulation of smooth muscle cell (SMC) active tone as well as collagen synthesis and removal in response to a flow perturbation, and associated changes in wall shear stress, and a pressure perturbation, and associated changes in wall stress.

Changes in circumferential and axial stress appear to be sensed directly by smooth muscle cells, which then change their gene expression profile (Bishop, 1998; Chiquet, 1999). Amongst the many different changes, increased wall stress leads to an increased smooth muscle cell production of ECM, particularly collagen (Sasamura et al., 2005). This increase in collagen production is driven by various stress-mediated biomolecules, including transforming growth factor-β, a potent stimulator of collagen synthesis (Ryan et al., 2003). Hence, if a pressure-induced increase in wall stress persists for a modest period, say days to weeks, the arterial wall will thicken due to increase in collagen production, both in the media and adventitia (Fridez et al., 2003; Matsumoto & Hayashi, 1994). Studies also indicate that excess stretch or stress suppresses matrix metalloproteinase (MMP) production and their effectiveness (Maeda et al., 2013), thus reducing collagen degradation. See Figure 1 for a schematic drawing of the above discussion.

**Figure 2.**
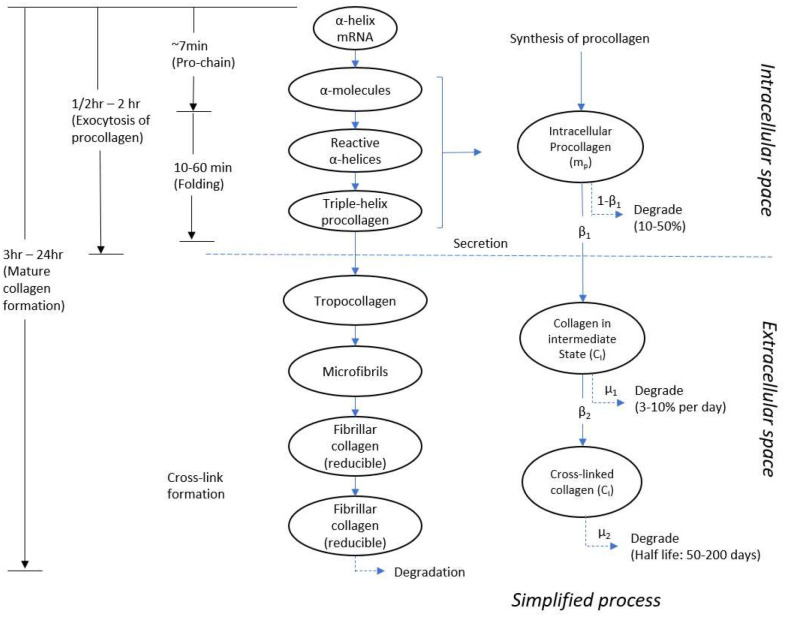
Schematic illustration of the production and removal of mature collagen. On the right-side, collagen production is simplified into three stages: newly synthesized procollagen molecules in the intracellular space as well as intermediate microfibrils and cross-linked collagen fibers in the extracellular space.

The active mechanosensitive processes within vascular cells depends in large part on the physical linkage of the ECM to the cytoskeletal structure (Davis & Hill, 1999; Liu & Lin, 2022). Transmembrane adhesion molecules, that is, integrins, plays a central role in this linkage, enabling cells both to mechano-sense and mechano-regulate their ECM in response to changes in mechanical stimuli (Bishop, 1998; Chiquet, 1999; Lehoux et al., 2006). Although the resulting mechano-transduction signals are complex and control a host of transcriptional changes, we focus on the turnover of fibrillar collagen, and in particular collagen I which constitutes about 60–70% of all vascular collagen (Sasamura et al., 2005). The synthesis, deposition, and degradation of fibrillar collagen represent a complex series of processes, summarized in Figure 2.

Following mechanotransduction, associated mRNAs within the nucleus are transported to cytoplasmic ribosomes (Laurent, 1987). The appropriate amino acids (over 1000 per chain) are then sequenced in a (G-X-Y)_n_ motif, where G is glycine, to form an α-chain. Three α-chains are combined to form procollagen, a triple (super) helix molecule. Prepared in the Golgi apparatus, the procollagen molecules are transported in vesicles that can coalesce at the cell membrane and be secreted in a mechano-regulated fashion. Once in the extracellular space, propeptides are cleaved from the ends and the tropocollagen molecules (or simply collagen, ~1.5 nm diameter and 300 nm long with a MW~300000 daltons) (Jawad & Brown, 2011; Nimni, 1992) are assembled to form micro-fibrils with a release of water molecules; subsequent aggregation and cross-linking of fibrillar collagen results in long interconnected fibers having diameters of microns.

Not all of the synthesized procollagen is secreted, however. About 10–50% (Goldberg, 2003; McAnulty & Laurent, 1987; Schubert et al., 2000) (in some estimates 10–20% (Berg, 1986) or 30–40% (Bienkowski et al., 1978)) is degraded inside the cell within minutes of synthesis. Such degradation may represent a type of quality control, ensuring that secreted molecules contain the precise chemical structure to ensure effective incorporation into structural fibers (Goldberg, 2003). The secreted procollagen that is converted into tropocollagen (Kao et al., 1979) aggregates within about 20 min (Gelman et al., 1997). There is a general claim that procollagen is converted into collagen around the moment of exocytosis (Berg, 1986). Daily, 3–10% of the extractable collagen is degraded (Sodek, 1976). From the remaining extractable collagen, about 1–8% per day is cross linked and converted into mature cross-linked collagen fibers (Niedermüller et al., 1977), which have a normal half-life from 50 days to 100 days (Gineyts et al., 2000; Nissen et al., 1978). According to Nimini (1992), formation of individual α-chains, assembly into a triple helix motif, and packaging and secretion to the extracellular space take on the order of ~7, ~8, and ~20 minutes, respectively. Not including time for gene transcription, and depending on the specific tissue and collagen type, it takes from about 30 minutes to 24 hours to produce collagen (Marchi & Leblond, 1983; Nimni, 1992). Although the extracellular cross-linking can occur quickly, the overall degree of cross-linking may change over longer periods (hours to weeks or months) as the fibers ‘mature’. For example, after chronic flow reduction in mice, a permanent change in arterial structure may be noticeable as early as three days in cases of sustained flow reductions, with changes persisting for weeks or more (Rudic et al., 2000).

Extracellular collagen can be degraded via two different pathways, intracellular or extracellular. The intracellular pathway may dominate under physiological conditions (Everts et al., 1996) and is accomplished via a phagocytotic ingestion by the cells. By contrast, the extracellular pathway appears to dominate in cases of disease, injury, and perhaps even perturbations in mechanical loading; it is affected via a variety of enzymes, including MMPs, serine proteases, and cysteine proteases. MMPs appear to be the major class of enzymes responsible for the degradation of collagen fibers in most cases of arterial G&R (Chase & Newby, 2003).

## 3. Biochemomechanical Model

We first present a chemical kinetics model for collagen production, assembly, and removal in Sec. 3.1, then summarize the constrained mixture model in Sec. 3.2. The biochemical model is coupled with the constrained mixture model to form a biochemomechanical model of vascular adaptation in Sec.3.3. Finally, simulations are presented in Sec. 3.4.

### 3.1. A Simplified biochemical model of collagen turnover

Although the chemical steps of collagen synthesis are now understood and detailed kinetics models could be linked directly to vascular G&R using parameter estimation, it is still difficult to estimate specific reaction rates between each step. Hence, rather than developing a detailed reaction model, with many unknown parameters, we simplify the reactions from intracellular procollagen production to mature collagen assembly via two steps that include four net parameters.

As shown in Figure 2, the extracellular collagen is classified into an intermediate state, which represents assembled microfibrils, and a final state, which represents fibrillar collagen with mature cross-links. Similar to prior work (Niedermüller et al., 1977), we consider first-order reactions, with an initial supply *m*_*p*_ scaled by *β*_1_ (fraction of procollagen that is released to the extracellular space), a conversion from intermediate to final parameterized by rate *β*_2_, and degradation in each of the two extracellular states parameterized by rates *μ*_1_ and μ_2_, respectively. The governing equations are thus:

(2) $$\frac{\partial C_{I}}{\partial t}=\beta_{1}m_{p}-\left(\beta_{2}+\mu_{1}\right)C_{I},$$

(3) $$\frac{\partial C_{F}}{\partial t}=\beta_{2}C_{I}-\mu_{2}C_{F},$$

where C_*I*_ and C_*F*_ are the molar concentrations of intermediate and cross-linked matured collagen. Values of *β*_1_, *β*_2_, *μ*_1_, and *μ*_2_ with their corresponding references are in Table 1. Measurement of collagen turnover without considering reutilization of isotopic precursors used to label the collagen can result in longer than actual turnover times (Laurent, 1987; Sodek & Ferrrier, 1988), hence actual rates may be greater than the values indicated in Table 1. The mean age of the mature collagen is thus given by $a_{\text{mean}}=\frac{1}{\mu_{2}}=100$ day, with *μ*_2_ constant (0.01 day^−1^).

Although degradation of mature fibers depends on additional variables, such as the number of cross-links of the fibers (both enzymatic and non-enzymatic, which typically change with aging), we only use the current state of stress and concentrations of enzymes (mainly MMPs) to model degradation. It appears, for example, that increasing the concentration of MMPs accelerates the rate of degradation while perturbing stress can alter the conformation of the collagen fibers and influence MMP efficacy (Ruberti & Hallab, 2005). We also assume that only the cross-linked mature collagen contributes to mechanical load bearing (i.e., strain energy function) in the biomechanical model.

### 3.2. Biomechanical model

The continuum theory of mixtures is well suited for describing the behavior of arteries (Ateshian & Humphrey, 2012; Humphrey & Rajagopal, 2002), though it is currently not possible to capture the chemical or mechanical contributions of all of the ECM components (there are on the order of 100 different proteins, glycoproteins, and glycosaminoglycans within the arterial wall). Hence, we focus on the three primary structurally significant constituents: elastin, collagen fibers, and smooth muscle cells (SMCs) (Bank et al., 1996; Figueroa et al., 2009). The actual complexity of arterial mechanobiology, in view of the large number of constituents that could be tracked, necessitates judicious choices that capture the salient responses of the mixture by following only the key constituents. Thus, let material properties at each place ***x***(*t*) in the mixture configuration *k*_*t*_(*B*) be modeled by assuming that, in a homogenized sense, multiple constituents co-exist within local neighborhoods and at each time *t*.

We denote constituents SMCs, collagen fibers (using a 4-fiber family model with angle *α*), and elastin via *i ϵ* [*m*, *k*, *e*], whereby we assume that SMCs are oriented in the circumferential direction, collagen has four families in axial, circumferential, and symmetric diagonal directions (*k* = *1*, *2*, *3*, *4*) and elastin is an amorphous material. We also assume the same mechanical properties for multiple families *k* of locally parallel collagen fibers. In this biomechanical model, the time variable *τ* is the time at which the newly deposited cross-linked collagen starts to carry stress. Multiple configurations, *k*_*R*_(*B*) and $\kappa_{n(\tau )}^{i}$, and their transformations from one to other configurations have been defined previously (Baek et al., 2007; Valentín et al., 2009) (Fig. 3a). Let the deformation of the *i*^*th*^ constituent that was produced at time *τ* be described by the linear transformation $F_{n(\tau )}^{i}(t)$:

(4) $$F_{n(\tau )}^{i}(t)=F(t)F{\left(\tau \right)}^{-1}G_{h}^{i}(\tau ).$$

For modeling arterial deformation during the G&R, we assume an axisymmetric thin wall with ***F***(*t*) is given by *diag*[*λ*_1_(*t*), *λ*_2_(*t*)], where *λ*_1_(*t*) = *l*(*t*)⁄*l*_ℎ_, *λ*_2_(*t*) = *r*(*t*)/*r*_*h*_, *l* is axial length, and *r* is the mean radius. $G_{h}^{i}(\tau )$ is the deposition stretch. $F_{n(\tau )}^{i}$, which can be written as $\text{diag}\left(\lambda_{n,1}^{i},\lambda_{n,2}^{i}\right)$, transforms vectors that belong to the tangent space at ***x***_*n*(*τ*)_ *ϵ k*_*n*(*τ*)_(*B*) to the tangent space at ***x*** *ϵ k*_*t*_(*B*) for the *i*^*t*ℎ^ constituent that was produced at time *τ* ≤ *t*. Again, following prior work (Baek et al., 2007; Valentín et al., 2009), the strain energy is given as

(5) $$w^{i}(t)=\int_{t-a_{max}^{i}}^{t}m_{R}^{i}(\tau )q^{i}(t,\tau )\Psi^{i}\left(F_{n(\tau )}^{i}(t)\right)d\tau ,$$

where $m_{R}^{i}(\tau )>0$ is rate at which new structural constituents *i* are produced at time *τ*, with $a_{max}^{i}$ the maximum age of constituent *i*. In this paper, any quantity that is denoted by (·)_*R*_ is expressed relative to a fixed reference configuration. The *q*^*i*^(*t*, *τ*) ∈ [0,1] is the fraction of constituent *i* that is produced at time *τ* and survives to time *t*. It is inspired by population dynamics (Thieme, 2003) and can be expressed as

(6) $$q^{i}(t,\tau )=exp\left(-\int_{\tau }^{t}\mu_{2}^{i}(s)ds\right).$$

Functional forms for $m_{R}^{i}(\tau )$ and $\mu_{2}^{i}(s)$ will be derived in the next section. Finally, Ψ^*i*^ is the strain energy of a constituent *i* per unit mass, which can be computed via

(7) $$\Psi^{m}=\frac{c_{1}^{m}}{2}\left({\left(\lambda_{n,1}^{m}\right)}^{2}+{\left(\lambda_{n,2}^{m}\right)}^{2}+\frac{1}{{\left(\lambda_{n,1}^{m}\right)}^{2}{\left(\lambda_{n,2}^{m}\right)}^{2}}-3\right)+\frac{c_{2}^{m}}{4c_{3}^{m}}\left(exp\left(c_{3}^{m}{\left({\left(\lambda_{n,2}^{m}\right)}^{2}-1\right)}^{2}\right)-1\right),$$

(8) $$\Psi^{k}=\frac{c_{2}^{c}}{4c_{3}^{c}}\left(exp\left(c_{3}^{c}{\left({\left(\lambda_{n}^{k}\right)}^{2}-1\right)}^{2}\right)-1\right)$$

(9) $$\Psi^{e}=\frac{c_{1}^{e}}{2}\left({\left(\lambda_{n,1}^{e}\right)}^{2}+{\left(\lambda_{n,2}^{e}\right)}^{2}+\frac{1}{{\left(\lambda_{n,1}^{e}\right)}^{2}{\left(\lambda_{n,2}^{e}\right)}^{2}}-3\right).$$

Because newly produced material could have a different material symmetry than that which is in place, the material parameters within Ψ^*i*^ can change with time. The newly produced material depends on the stress and the history of the deformation. The constitutive relation for the Cauchy membrane stress (force per deformed length) is:

(10) $$T=\frac{1}{J}F\frac{\partial w}{\partial F^{T}}\to T_{11}=\frac{1}{\lambda_{2}}\frac{\partial w}{\partial \lambda_{1}},T_{22}=\frac{1}{\lambda_{1}}\frac{\partial w}{\partial \lambda_{2}}$$

where *J* = *λ*_1_*λ*_2_. Denoting the membrane stress due to vascular smooth muscle tone by ***T***_*act*_, the total Cauchy membrane stress of a vasoactive vessel is:

(11) $$T=T_{\text{pass}}+T_{act},$$

where *T*_*pass*_ is the passive Cauchy membrane stress and *T*_*act*_ is computed, by slightly modified constitutive relations from previous work (Baek et al., 2007; Rachev & Hayashi, 1999):

(12) $$T_{act}=S\lambda_{2}^{act}f\left(\lambda_{2}^{act}\right)e_{2}⊗e_{2},$$

(13) $$S(t)=2S_{\text{basal}}\frac{M_{R}^{m}(t)}{M_{R}^{m}(0)}\left(1+\text{tanh}\left(-k_{ton}\left(\frac{\tau_{w}(t)}{\tau_{wh}}-1\right)-\text{ln}\left(\frac{3}{2}\right)\right)\right)\text{, }$$

(14) $$f\left(\lambda_{2}^{act}\right)=1-{\left(\frac{\lambda_{M}-\lambda_{2}^{act}}{\lambda_{M}-\lambda_{0}}\right)}^{2},$$

where *S*_*basal*_ is the basal vasoactive tone, $M_{R}^{m}$ is the total mass of SMCs, *k*_*ton*_ is a scaling constant, and *τ*_*w*ℎ_ is the homeostatic wall shear stress. Moreover, *λ*_*M*_ and *λ*_0_ are the stretches at which the contraction is maximum and zero, respectively, and $\lambda_{2}^{act}$ is an active stretch that is computed from (Baek et al., 2007):

(15) $$\frac{dr^{act}}{dt}=K_{act}\left(r-r^{act}\right),\;\lambda_{2}^{act}=\frac{r}{r^{act}}$$

where *K*_*act*_ is constant.

Finally, the normal Cauchy stress equations in Eq. (1) can be re-written as:

(16) $$\sigma_{1}=\frac{T_{11}}{h},\;\sigma_{2}=\frac{T_{22}+S\lambda_{2}^{act}f\left(\lambda_{2}^{act}\right)}{h}.$$

### 3.3. Biochemomechanical model

In sections 3.1 and 3.2, we separately presented the biochemical and biomechanical models. To integrate these models into a single biochemomechanical model, we need a common computational configuration. Let *k*_*R*_(*B*) be a fixed reference configuration (at reference position ***X***) that is mapped into in vivo configurations (e.g., *k*_*t*_(*B*)) at different times during G&R. The use of fixed-reference configuration allows us to use a material description when modeling both the kinetics of mass turnover and changes in strain energy without considering volume or area changes.

We let the synthesis rate of intracellular procollagen and proliferation of SMCs, *m*_*pR*_(t) (Figure 2, Eq (2)) be given by a stress-dependent scalar function depending on time *t*. We also consider different rates of collagen production by the two primary cell types, fibroblasts in the adventitia and smooth muscle cells in the media:

(17) $$m_{pR}^{k}(t)=\frac{M_{R}^{c}(t)}{M_{R}^{c}(0)}\left(K_{\sigma }^{c}\left(\frac{\sigma^{k}(t)}{\sigma_{h}}-1\right)-K_{\tau_{w}}^{c}\left(\frac{\tau_{w}(t)}{\tau_{wh}}-1\right)+m_{p}^{k}(0)\right),$$

where *σ*_ℎ_ is the homeostatic normal stress, and *K*_*σ*_ and $K_{\tau_{w}}$ are gain-type parameters; $m_{pR}^{k}(t)$ is the rate of procollagen mass production and $M_{R}^{c}(t)$ is collagen mass. In Eq. (17), the ratio $M_{R}^{c}(t)/M_{R}^{c}(0)$ comes from an assumption that the total cell numbers is proportionally increased during the growth and each cell produces the procollagen (Baek et al., 2005).

After synthesis via $m_{pR}^{k}(t)$, a change in intermediate collagen, $C_{IR}^{k}$, is given by a differential equation,

(18) $$\frac{dC_{IR}^{k}(t)}{dt}=\beta_{1}m_{pR}^{k}(t)-\left(\beta_{2}+\mu_{1}\right)C_{IR}^{k}(t).$$

In order to utilize the reaction equations from intermediate to mature collagen, we consider the production of matured collagen, meaning conversion from the intermediate collagen, which is soluble, to cross-linked collagen fibers, *k*, where the mass production rate $m_{R}^{k}(\tau )$ at time τ is given by

(19) $$m_{R}^{k}(\tau )={\left(MW\right)}^{c}\beta_{2}C_{IR}^{k}(\tau ),$$

where *MW*^*c*^ is the molecular weight of collagen. Written in this way, information from the ‘reaction kinetics’ can be incorporated directly within the mixture formulation, thus providing additional guidance on reasonable constitutive relations for constituent production and removal. The mass of matured cross-linked collagen fiber family *k* (and the total mass of collagen, *c*) per unit reference area is given by

(20) $$M_{R}^{k}(t)=\int_{t-a_{max}}^{t}m_{R}^{k}(\tau )q^{k}(t,\tau )d\tau ,\;M_{R}^{c}(t)=\sum_{k=1}^{4}M_{R}^{k}(t).$$

Scalar measures of wall stress and thickness in the collagenous fiber families are given by:

(21) $$\sigma^{k}(t)=\frac{\left|T^{c}y^{k}(t)\right|}{h^{c}(t)},\;h^{c}(t)=\frac{M_{R}^{c}(t)}{\left(1-\phi_{f}\right)\rho \lambda_{1}\lambda_{2}},$$

where ***T***^*c*^ = **∑**_*k*_***T***^*k*^, ***y***^*k*^ is unit vector in the direction of collagen family *k*, and *ϕ*_*f*_ is the mass fraction of fluid in the vessel (70%). Although the relative percentages and mechanical properties of elastin and collagen change with age (Seyedsalehi et al., 2015), we consider adaptations that occur over much shorter time scales. Hence, we take the baseline state as homeostatic.

The rate of degradation $\mu_{2}^{i}(t)$ can be given as a constitutive function of stress. It is also related to the relative tension in collagen fiber *k*, as, for example (Baek et al., 2007):

(22) $$\mu_{2}^{k}(t)=K_{\mu 1}^{k}+K_{\mu 2}^{k}{\left(\zeta^{k}(t)-\zeta_{c}^{k}\right)}^{2},$$

(23) $$\zeta^{k}(t)=\frac{\frac{\partial w^{k}}{\partial \lambda_{n(\tau )}^{k}}\left(\lambda_{n(\tau )}^{k}(t)\right)}{\frac{\partial w^{k}}{\partial \lambda_{n(\tau )}^{k}}\left(G_{h}^{k}\right)}.$$

We assume that the tissue has been in a homeostatic state for a long time prior to *t* = 0. Thus, the condition for a steady state for *k*^*t*ℎ^ collagen family is (from Eq. (2), (3) and (19), and observing that $M_{R}^{k}(t)={\left(MW\right)}^{c}C_{FR}^{k}(t)$),

(24) $$m_{R}^{k}(0)=m_{h}^{k}=\mu_{2}^{k}M_{R}^{k}(0),$$

(25) $$C_{IR}^{k}(0)=\left(\frac{1}{\beta_{2}^{c}}\right)\frac{\mu_{2}^{k}M_{R}^{k}(0)}{{\left(MW\right)}^{c}},$$

(26) $$m_{p}^{k}(0)=\left(\frac{\beta_{2}^{C}+\mu_{1}^{c}}{\beta_{1}^{C}\beta_{2}^{c}}\right)\frac{\mu_{2}^{k}M_{R}^{k}(0)}{{\left(MW\right)}^{c}}.$$

It is noteworthy that if a step-change in sustained flow or blood pressure occurs, the above conditions Eq. (24–26) do not hold during the transition; however, if the sustained flow or pressure is long enough, these steady-state conditions return to a new homeostatic state.

Similarly, SMCs mass will be computed from,

(27) $$m_{R}^{m}(t,\tau )=\frac{M_{R}^{m}(t)}{M_{R}^{m}(0)}\left(K_{\sigma }^{m}\left(\frac{\sigma^{m}(\tau )}{\sigma_{h}}-1\right)-K_{\tau_{w}}^{m}\left(\frac{\tau_{w}(\tau )}{\tau_{wh}}-1\right)+m_{R}^{m}(0)\right),$$

where $m_{R}^{m}(t,\tau )$ is the turnover rate of SMCs and $M_{R}^{m}(t)$ is the total mass of SMCs at time *t*. The rate of loss of smooth muscle cells (apoptosis) is assumed to be constant, $\mu_{2}^{m}=K_{\mu 1}^{m}=0.01{\;day}^{-1}$. Similar to Eq.(24), $m_{R}^{m}(0)=\mu_{2}^{m}M_{R}^{m}(0)$. The scalar measure of the stress is set to:

(28) $$\sigma^{m}(t)=\frac{T_{22}+S\lambda_{2}^{act}f\left(\lambda_{2}^{act}\right)}{h^{m}},\;h^{m}(t)=\frac{M_{R}^{m}(t)}{\left(1-\phi^{f}\right)\rho \lambda_{1}\lambda_{2}}.$$

### 3.4. Simulations

It has been shown previously that 2D (i.e., membrane) models can capture many salient features of arterial G&R (Gleason et al., 2004; Kim et al., 2020; Zhou et al., 2015), hence we use the same approach here. For the simulation, we assume an idealized circular-cylindrical vessel that is uniform along the axial direction without axial extension during the G&R, in which the mean radius *r* is the only independent variable with respect to time *t* during the simulation. Dimensions and corresponding stresses change when the pressure, *P*, or volumetric flow rate, *Q* are perturbed from homeostatic levels. Depending on the duration (Δ*t*) and magnitude of the perturbations (*δP* or *δQ*), the vascular dimensions (diameter (*t*), and wall thickness ℎ(*t*)) and stresses evolve in time (Figure 3).

**Figure 4.**
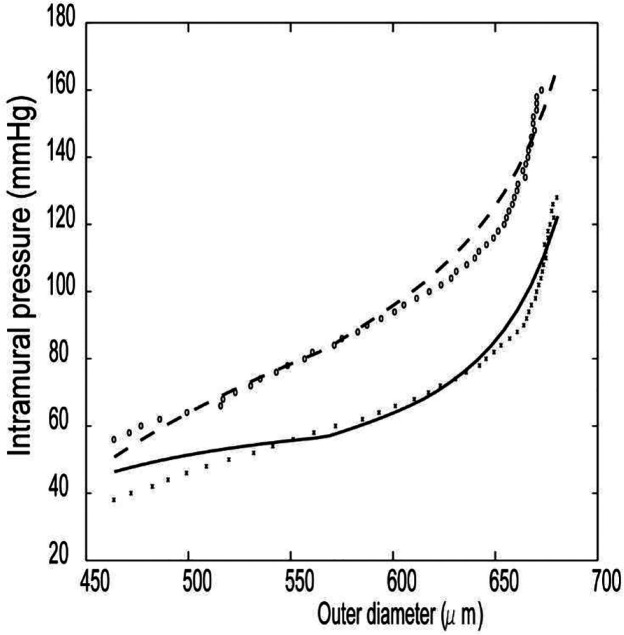
Intramural pressure (mmHg) vs outer diameter (μm) for a mouse carotid artery (Gleason et al., 2008). The passive and active parameters for arterial model were estimated from passive (_* * *_) and active (o o o) experimental data. The fit to data with the model is shown by the solid (passive) and dashed (active) curves.

**Figure 5.**
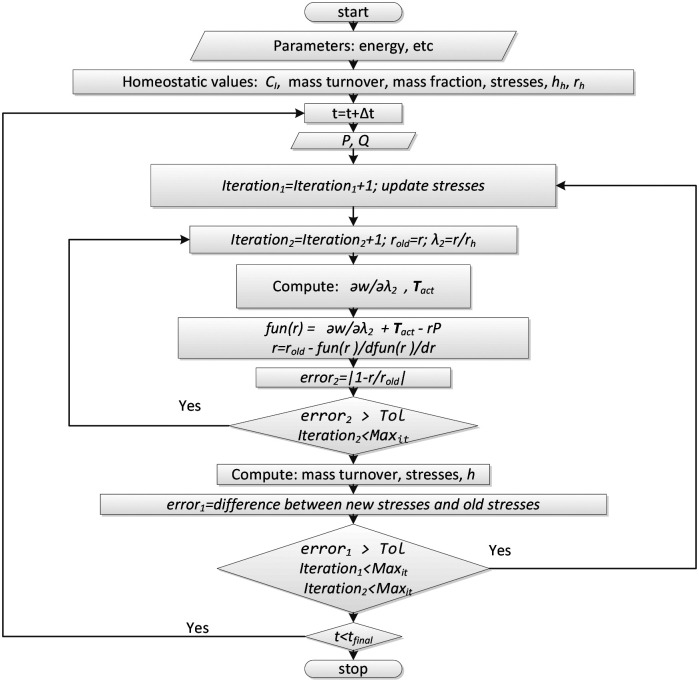
Flow chart indicating the solution procedure for different variables, given prescribed values of pressure (P) or flow rate (Q). The Newton-Rapson iteration that is used to determine the mean radius, r, at each time step, Δt, is the nested internal loop.

Parameters used in the simulation are listed in Table 2. Briefly, dimensions (*r*_*oh*_, *h*_*h*,_ *α*_ℎ_), parameters for the strain energy ($G_{h}^{c}$, $G_{h}^{m}$, $G_{1}^{e}$, $G_{2}^{e}$, $c_{1}^{e}$, $c_{2}^{c}$, $c_{3}^{c}$, $c_{1}^{m}$, $c_{2}^{m}$, $c_{3}^{m}$) and smooth muscle tone (*λ*_*M*_, *λ*_0_, *S*_*basal*_) are determined by optimization and curve fitting of both passive and active mechanical behavior of a mouse carotid artery data (Gleason et al., 2008), where the homeostatic mass fractions ($\phi_{0}^{e}$, $\phi_{0}^{m}$, *ϕ*^*f*^_, $\phi_{0}^{k}$_), stresses (*σ*_ℎ_, *τ*_ℎ_), and pressure (*P*_ℎ_) are assumed to be constants based on the references in Table 2. For the optimization, a penalty method is used to set ranges of pre-stretch values for each constituent to be bound using prior arterial G&R simulations (Seyedsalehi et al., 2015) and material parameters to be equivalent for the four collagen fiber families. Figure 4 shows optimization results for intramural pressure vs. outer diameter. Then, assuming that blood pressure and volumetric flow are independent input variables, the artery is assumed to be in the homeostatic state at and before *t* = 0; this state is then perturbed by either changes in the pressure or flow from homeostatic values. We then compute changes in the other arterial parameters as the time progresses. Using Eq. (10) and (11), the mean blood pressure and circumferential membrane stress for a vasoactive vessel are calculated from the equilibrium equation in the circumferential direction:

(29) $$Pr=T_{22}+S\lambda_{2}^{act}f\left(\lambda_{2}^{act}\right),$$

where pressure (*P*) is assumed to be known. After each time step (*t* + Δ*t*), we determine the radius using Newton-Rephson iteration, where the solution procedure is shown in Figure 5.

## 4. Results

### 4. 1 Stress-mediated adaptation

**Figure 6.**
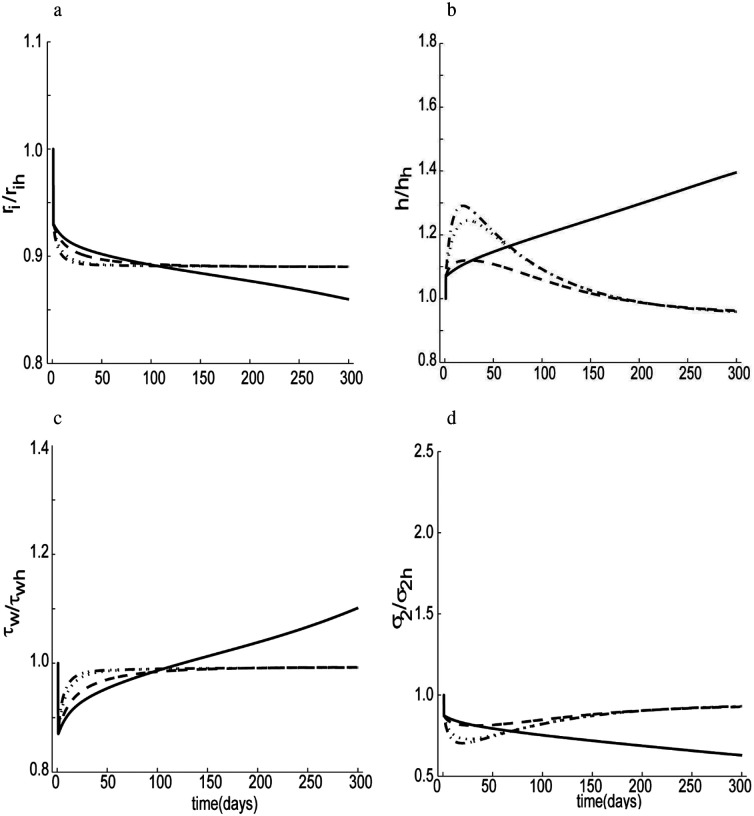
Predicted changes in dimension and stress in response to a 30% step decrease in volumetric flow rate as a function of wall stress-sensitivity: a) internal radius, b) arterial thickness, c) wall shear stress and d) circumferential stress. Results are shown for different vales of key mechano-regulation parameters: $K_{\sigma }^{i}=0$ (solid), $K_{\sigma }^{i}=2$ (dashed), $K_{\sigma }^{i}=6$ (dotted), $K_{\sigma }^{i}=10$ (dotted-dashed), and $K_{\mu 1}^{k}=K_{\mu 2}^{k}=0.01$, $K_{\tau_{w}}^{c}=K_{\tau_{w}}^{m}=52$, $k_{ton}=\frac{K_{\tau_{w}}^{c}}{3}$, μ_1_ = 0.1, β_2_ = 0.2. Clearly, mechano-regulation is needed for a homeostatic response.

As noted earlier, when the blood flow rate is reduced over a long period, the vessel geometry tends to adapt. We can investigate effects of changes in stress on these changes by varying the stress sensitivity parameters: $K_{g}^{c}$ and $K_{g}^{m}$ (see Eq. (17) and (27)). Many experimental studies have shown that wall shear stress tends to return close to targeted homeostatic values in response to modest changes in flow and similarly for wall stress (Hu et al., 2007; Humphrey, 1992). Hence, given information on the flow perturbation (for example, a 30% reduction in volume flow rate at a constant pressure, as shown in Figure 6), then, using Eq. (1), we can predict the final dimension of the artery, i.e., $\underset{t\to \infty }{\text{lim}}\frac{\tau_{w}}{\tau_{wh}}=\underset{t\to \infty }{\text{lim}}\frac{\sigma_{2}}{\sigma_{2h}}=1$, with *P* = *γP*_*h*_ and *Q* = *εQ*_*h*_, yields $\frac{r_{i}}{r_{ih}}=\frac{h}{\gamma h_{h}}=\varepsilon^{1/3}$, which for our case results in $\frac{r_{i}}{r_{ih}}=\frac{h}{h_{h}}=0.89$ for *γ* = 1 and *ε* = 0.5. Our simulation complies with this ideal prediction to different degrees depending on the value of stress sensitivity parameters. When mass production was assumed to be independent of normal stress (i.e., $K_{\sigma }^{c}=K_{\sigma }^{m}=0$), the internal radius adapted slowly and reduced beyond the expected ideal value, Figure 6(a), indicative of a weak luminal radius regulation. The corresponding shear stress, Figure 6(c), also passed beyond its homeostatic level and continued to increase. Without normal stress-regulation, wall thickness increased excessively, Figure 6(b). Thus, the circumferential stress similarly did not achieve its homeostatic value, as seen in Figure 6(d), but instead continued to fall as the simulation progressed. By contrast, when the strength of the mechano-regulation increased (i.e. increasing values of $K_{\sigma }^{c}$ and $K_{\sigma }^{m}$), the internal radius and shear stress returned to their corresponding homeostatic levels quicker. Conversely, higher values of $K_{\sigma }^{c}$ and $K_{\sigma }^{m}$ resulted in a large overshoot in thickness change and similarly a large undershoot in hoop stress change.

**Figure 7.**
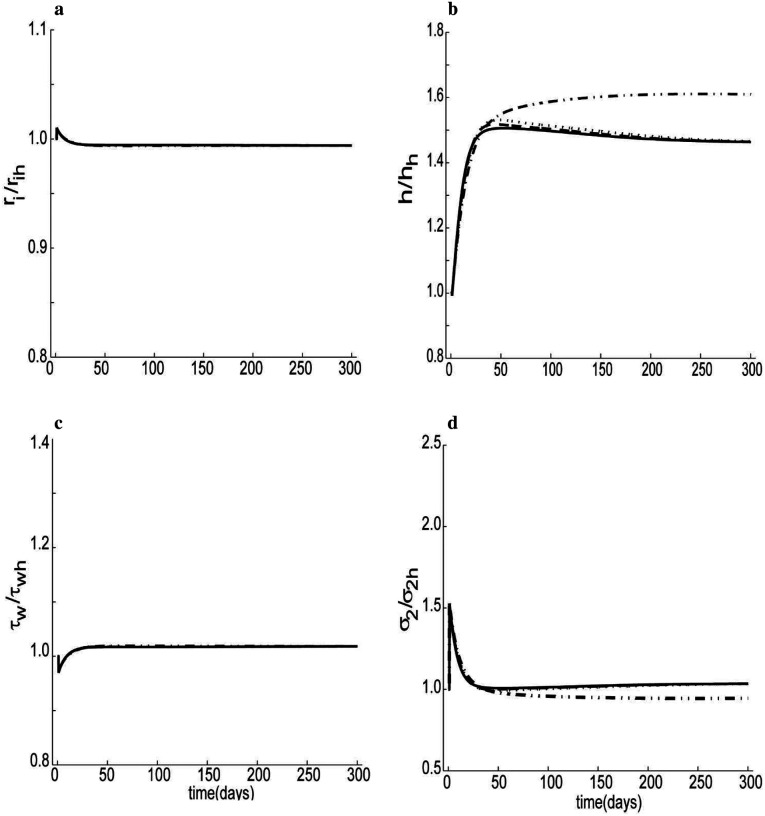
Similar to Figure 6 except for a step 50% increased pressure: a) internal radius, b) arterial thickness, c) wall shear stress and d) circumferential stress, with μ_1_ = 0.0, β_2_ = 0.001 (dotted-dashed); μ_1_ = 0.0, β_2_ = 0.05(dotted); μ_1_ = 0.05, β_2_ = 0.05(dashed); μ_1_ = 0.1, β_2_ = 0.2(solid); all with $K_{\sigma }^{c}=K_{\sigma }^{m}=2$, $K_{\mu 1}^{k}=K_{\mu 2}^{k}=0.01$, $=K_{\tau_{w}}^{c}=K_{\tau_{w}}^{m}=3k_{ton}=52$.

Similarly, when the pressure was increased 50% above its homeostatic value (at a constant volumetric flow rate), the simulation suggested that internal radius, wall shear stress, and hoop stress returned close to the homeostatic level with reasonable values of the stress-mediated gain parameters (Figure 7(a, c and d)).

### 4.2. Parametric study of collagen biochemical kinetics

**Figure 8.**
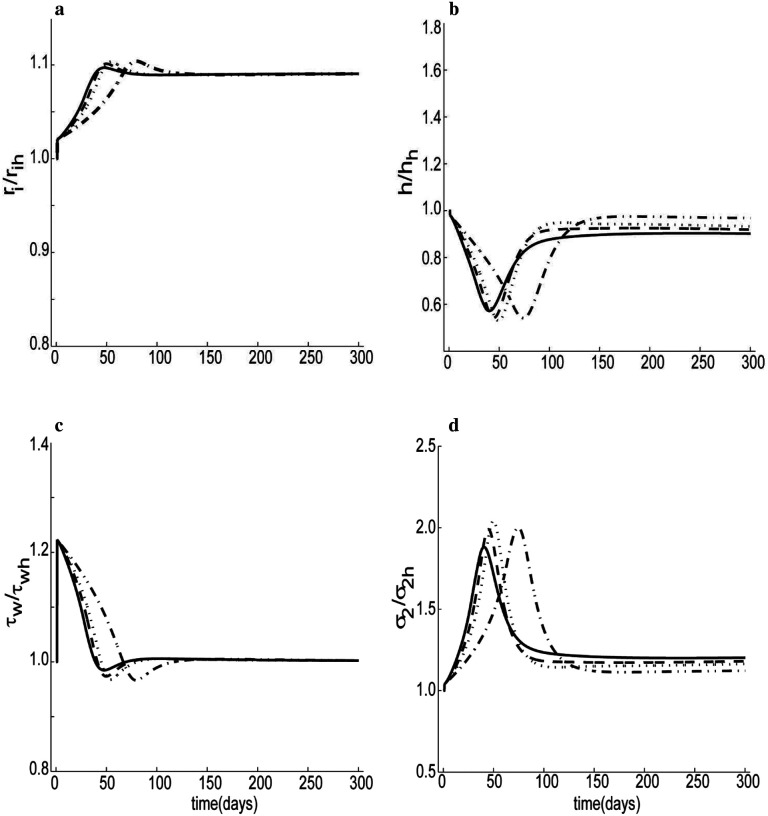
Simulations for a 30% step increase in volumetric flow rate: a) internal radius, b) arterial thickness, c) wall shear stress and d) circumferential stress, with μ_1_ = 0.0, β_2_ = 0.001(dotted-dashed); μ_1_ = 0.0, β_2_ = 0.05 (dotted); μ_1_ = 0.05, β_2_ = 0.05(dashed); μ_1_ = 0.1, β_2_ = 0.2(solid), all with $K_{\sigma }^{c}=K_{\sigma }^{m}=3$, $K_{\mu 1}^{k}=K_{\mu 2}^{k}=0.01$, $K_{\tau_{w}}^{c}=K_{\tau_{w}}^{m}=3k_{ton}=52$.

Intermediate collagen removal rate *μ*_1_ and cross-linking collagen rate *β*_2_ are directly related to temporal dynamics associated with the transition from intermediated to cross-linked collagen. As it should have, thickness increased 50% above its original level in response to a 50% increase of pressure (Figure 7(b)), though sensitive to the values that defined the strength of mechano-regulation and collagen turnover. As the combination of *β*_2_ and *μ*_1_was increased from 0.001 to 0.3, the thickness approached to its preferred value faster. Luminal radius and shear stress were relatively insensitive to collagen turnover when pressure was increased and flow rate was constant. We also studied the sensitivity of luminal changes to a wide range of blood volumetric flow rates with mechano-regulation included. When the volumetric flow rate increased by 10% or 30% (over a relatively short period), the internal radius increased by about 1.8% and 2.1%, respectively, whereas if flow decreased by the same magnitude, the internal radius decreased by about 4.5% and 8.2%, respectively (supplementary figure, Figure S1). During early changes (e.g., within one day in Figure S1), we see that initial luminal control depends largely on the vasodilatory versus vasocontractility capacity. This finding is qualitatively consistent with previous reports, including those for cerebral arteries (Valentín et al., 2009). Figure 8 shows a case for a 30% increase in flow over 300 days. An ideal adaptation, where wall shear stress returns to its hemostatic level, should yield a final value of the internal radius of $\frac{r_{i}}{r_{ih}}=1.09$. Figure 8(a) shows a result consistent with an ideal adaptation but with a decreasing settling time for increases in collagen turnover constants (starting from about three months for *μ*_1_ + *β*_2_ = 0.001 to about a month for *μ*_1_ + *β*_2_ = 0.3). The peak values for internal radius shift to the left and decrease in magnitude as turnover constants increase. Although the final simulated thickness nearly equals the homeostatic value, Figure 8(b), the transient undershoots are very sensitive to the collagen turnover constants. As collagen turnover increases, the magnitude of the undershoot reduces and shifts towards the left. Figure 8(c and d) shows corresponding variations in the stresses for different collagen turnover constants. Overall, the different panels in Figure 8 show that, as collagen turnover increases, the adaptation of the artery to a flow perturbation is faster.

### 4.3. Intrinsic changes in smooth muscle tone

**Figure 9.**
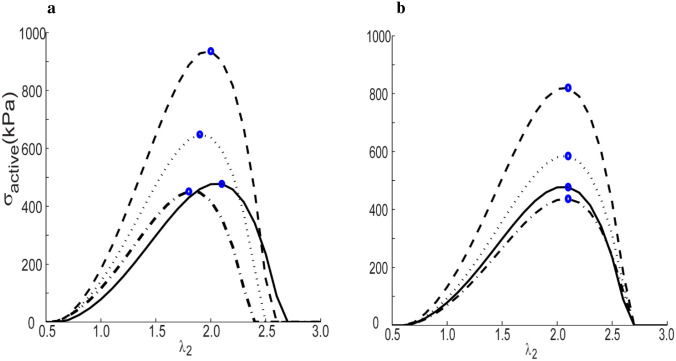
Active stress (kPa) vs normalized muscle fiber stretch at different days for a) 30% decreased volume flowrate and b) 50% increased pressure ($K_{\sigma }^{c}=K_{\sigma }^{m}=3$, $K_{\mu 1}^{k}=K_{\mu 2}^{k}=0.01$, $K_{\tau_{w}}^{c}=K_{\tau_{w}}^{m}=3k_{ton}=52$, μ_1_ = 0.1, β_2_ = 0.2), with results shown at 0 (solid), 7 (dashed), 14 (dotted), or 300 days (dotted-dashed). The circles indicate local maxima for the respective curves.

Intrinsic changes in smooth muscle tone can also play important roles over different periods. Inspired by prior studies (Valentín et al., 2009), we simulated variations in active stress, $\sigma_{act}=\frac{T_{act}}{h_{m}}$, for different circumferential stretches on different days, as G&R progresses (Figure 9). For a 30% decrease in volumetric flow rate, the changes in muscle tone are relatively large over a period of days (Figure 9(a)). As the circumferential stretch (λ_2_) increases from 0.65, the active stress increases and the peak active value is attained at stretches of 2.1, 2.0, 1.9, and 1.8 for muscle mass fractions $\left(\frac{M_{R}^{m}(t)}{M_{R}^{m}(0)}\right)$ taken at basal, 7, 14, and 300 days, respectively. Clearly, there is a left-ward shift of the peak active stress as the artery adapts to perturbations in flow. On the other hand, for a 50% increase in transmural pressure, the simulation suggested, Figure 9(b), that the peak active stress occurs at constant circumferential stretch of 2.1, while the contribution of active stress declines rapidly, from 820 kPa at day 7 to 437 kPa at day 300. The active stretch range in Figure 9 is similar to a prior study (Butler et al., 2011) and the magnitude of active stress is comparable to another study (Price et al., 1983)

## 5. Discussion

Although prior G&R models have described and predicted changes in response to altered blood pressure and flow rate over long periods (more than 6 months or years) (Baek et al., 2007; Valentín et al., 2009), those models do not incorporate matrix intrinsic (shorter) timescales. Hence, we replaced a simple macroscopic mass turnover rate used previously with a biochemical collagen turnover process: from synthesis rates of pro-collagen to cross-linked collagen fibers plus rates of removal of intermediate collagen within the extracellular space. This biochemical rate model still allows constituent turnover to depend on mechanical stress, with the associated continuum equilibrium problem enabling us to track temporal changes in geometry and stress during adaptations to changes in hemodynamics.

Overall, this new biochemomechanical model successfully predicted salient aspects of arterial G&R, including adaptations over periods from weeks to a few months. Outcomes from the simulations tend to be intuitive and consistent with observations; yet, the complex couplings among shear and normal stress-mediated G&R as well as differential synthesis and degradation rates renders it difficult to intuit the underlying reasons for any overall (mal)adaptation. A computational model can be useful in this regard. For example, one can assess differences in increased mass fractions of collagen and smooth muscle by studying parametrically the relative contributions of $K_{\sigma }^{c}$ or $K_{\sigma }^{m}$ to G&R (Eq. (17), (27)). Similarly, one can study potential effects of differential degradation rates for intermediate and mature collagen or conversions of intermediate into mature collagen (this can be modeled by varying *β*_2_ or μ_1_ in Eq. (2)), noting that early and long-term mechanisms may differ (Figure 7).

The simulation results of Figure 6 and Figure 8 show temporal predictions in vascular adaptation for two cases: a step-decrease versus a step-increase in blood flow, respectively. For decreases in flow the luminal area decreases while wall thickness increases for the first few months but then returns toward the homeostatic level within three months. Conversely, for step increases in blood flow, the simulation predicts an increased luminal area, a reduction in wall thickness, and associated increases in shear and normal stresses. The latter two return towards the homeostatic level within four months. Differences in response to decreases and increases in flow stem primarily from the differential effects of NO and ET-1 on collagen synthesis. Extreme transient reductions in wall thickness during increased flow could result in hyper-perfusion syndrome, which could lead to hemorrhage (Abou-Chebl et al., 2004). By contrast, when pressure increased, Figure 7, the simulation showed an increase in wall thickness with a preserved luminal area, consistent with most previously reported results (Fridez et al., 2003; Valentín et al., 2009). An increased thickness can help restore wall stress toward normal but it can also increase the structural stiffness that influences the hemodynamics, which may not be favorable (Anderson, 2006; Humphrey et al., 2016).

**Figure 10.**
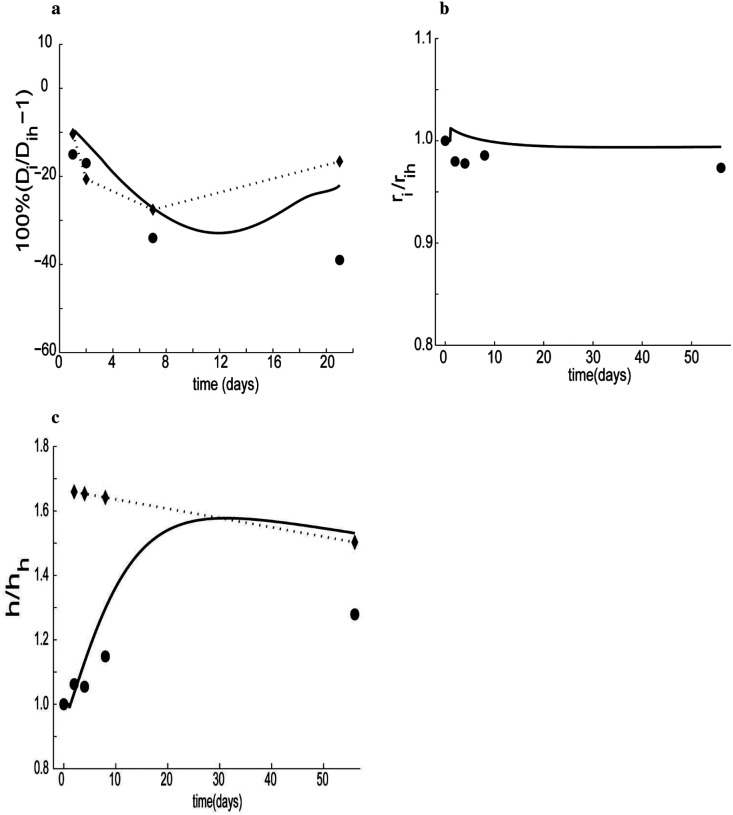
a) Simulated changes in internal diameter (solid line), ideal adaptation (diamond-dotted), and experimental data (circles) reported by Sluijter et al.(2004b); b) Simulated changes in internal diameter (solid line), and experimental data (circles) reported by Fridez et al.(2003) and c) corresponding thickness: simulated (solid), experimental (circles), and ideal adaptation (diamond-dotted), all with ($K_{\sigma }^{c}=K_{\sigma }^{m}=3$, $K_{\mu 1}^{k}=K_{\mu 2}^{k}=0.01$, $K_{\tau_{w}}^{c}=K_{\tau_{w}}^{m}=3k_{ton}=52$, μ1 = 0.1, β_2_ = 0.2).

Simulated parametric studies should be checked against experimental data whenever possible; in cases where data are not available, such models can guide the experimental plan. Figure 10(a) compares simulated results for diameter and wall shear stress against data from carotid ligation studies. The measured blood volumetric flow rate over 21 days was used as input in the simulation, which yielded results comparable to that which was reported experimentally (Sluijter et al., 2004b). Because there was a lack of pressure measurements over the reported period, we assumed that pressure remained constant. The ideal luminal change (dotted line) is smaller than the experimental report, and our simulation predicts values that are in between the two sets of values after 21 days.

The simulated change in shear stress is smaller than the reported change. Deviations between the reported experimental values and our simulations could be due to additional effects, including inflammation, that are not included in the current model and remaining unknown differences across species (e.g., rabbits versus mice). Comprehensive data are clearly needed to better inform the model. We also used a variable pressure, Figure 10(b and c), as input over 56 days. The simulation result is similar to the reported findings. Even though blood pressure increased more than 65%, the internal diameter stayed almost constant, slightly below the homeostatic level as per the reported experiment (Fridez et al., 2003). Compared to the prior data (Fridez et al., 2003), our mathematical model overestimated the chronic thickness of the vascular wall, which may have resulted from an excess generation of collagen or smooth muscle mass. Assuming the volumetric flow rate was constant, our simulation matches the ideal adaptation after about a month. More experimental data are needed, however, to verify the true source of the deviation. When pressure was increased by 50%, the circumferential stress reached its homeostatic level within 14–16 days, and its chronic level (which is slightly lower than the homeostatic value) within 45 days. This result is consistent with prior findings (Hu et al., 2007), wherein circumferential stress returned to the homeostatic level as early as 2 weeks after aortic coarctation to induce hypertension.

Although the biochemistry of intracellular and extracellular collagen turnover is well-documented and collagen cross-linking and removal rates are known reasonably well (summarized in Table 1), reported ranges are wide and differences in rates may likely vary with various physiological (e.g., age) and pathological (e.g., hypertension) changes, hence complicating predictions of temporal changes during vascular adaptations, with or without pharmaceutical intervention. In particular, the stability of intermediate cross-linked collagen depends on subject age and genetics (Bailey & Shimokomaki, 1971; Eyre & Glimcher, 1972; Robins et al., 1973) and the kinetic parameters *β*_2_ or μ_1_ should be related to specific cases.

Some of the deviations of our results from reported experiments could also be ascribed to limitations of our model. For example, we did not include mechanical damage to elastin, which could happen in both marked increases in flow and pressure (Chow et al., 2013). We also did not account for possible viscoelastic effects, noting that viscoelastic stress relaxation and G&R stress recovery could appear similar experimentally. Given the time scales involved, however, we feel that viscoelasticity should be negligible. We also used a 2D (membrane) rather than 3D model of the wall, which has been shown previously to be able to capture salient changes in geometry and structural stiffness but not details such as residual stress (Karšaj & Humphrey, 2012). Given that residual stress related opening angle data were not available in the experimental reports we used, this was not a key issue although 3D models could certainly offer greater insight. Finally, the biochemical model was simplified to only two steps and we neglected possible stress mediated dynamics of the proteolytic enzymes. Again, additional data will be needed to increase model sophistication. We submit, however, that the present model both recovers prior simulation results based on phenomenological descriptors and enables greater information on collagen turnover to be incorporated. The next major challenge will be to link such changes in turnover to specific stress-mediated changes in gene expression.

## Supplementary Material

1

## Figures and Tables

**Figure 3.**
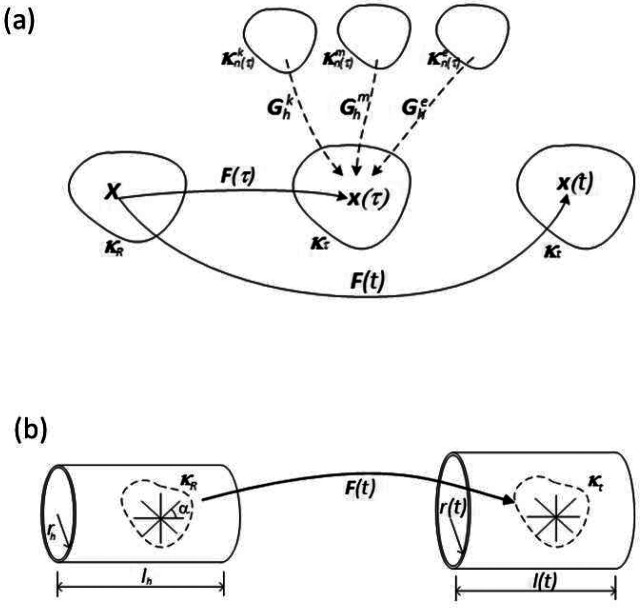
(a) Important configurations and their mappings: The reference configuration k_R_(B) is used for a computational reference as an initial geometry in homeostatic conditions. The natural configurations, $\kappa_{n(\tau )}^{i}$ are stress-free configurations of constituent i at time τ. (b) Deformation of an arterial segment from the idealized thin wall in a homeostatic condition to the deformed wall at t.

**Figure 3.**
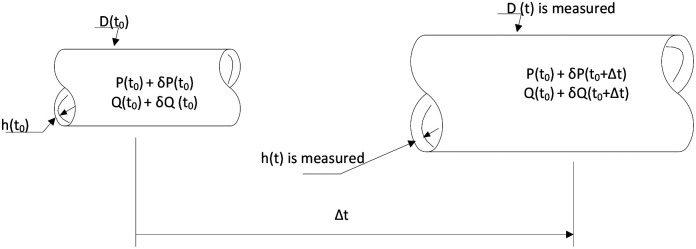
An idealized model for growth and remodeling of an artery in response to perturbed hemodynamics.

**Table 1.** Ranges of biochemical parameters estimated from the literature

Para.	Ranges	Units	Reference
*β*_1_	0.5–0.9		(Berg, 1986)
*β*_2_	0.01–0.2	day^−1^	(Laurent, 1987; Niedermüller et al., 1977; Sodek & Ferrrier, 1988)
*μ*_1_	0.03–0.1	day^−1^	(Niedermüller et al., 1977)
*μ*_2_	0.01–0.02	day^−1^	(Gineyts et al., 2000)
*MW*	4.981617×10^−22^	Kg	(Lewis et al., 2008)
*a*_*max*_	350	Day	(Gineyts et al., 2000; Nissen et al., 1978)

**Table 2.** Parameter Estimation of Mouse Carotid Artery (When parameters were not prescribed from literature, values were estimated via regression of data)

Para.	Mouse Carotid Artery	Units	Reference
*r*_*oh*_	0.328	mm	(Dye et al., 2007; Gleason et al., 2008)
*h*_*h*_	21.2	μm	(Dye et al., 2007; Gleason et al., 2008)
$G_{h}^{c}$	1.07		-
$G_{h}^{m}$	1.25		-
$G_{1}^{e}$	2.19		-
$G_{2}^{e}$	1.64		-
*σ*_*h*_	120	kPa	(Humphrey, 1992)
*τ*_*h*_	1.50	Pa	(Kamiya et al., 1984; Tronc et al., 2000)
$\phi_{0}^{e}$	0.06		(Dye et al., 2007; Gleason et al., 2008)
$\phi_{0}^{m}$	0.09		(Dye et al., 2007;Gleason et al., 2008)
*ϕ* ^*f*^	0.70		(Dye et al., 2007; Gleason et al., 2008)
$\phi_{0}^{k}$	0.15*{0.111,0.0349,0.427,0.427}		-
*P*_*h*_	102	mmHg	(Dye et al., 2007; Gleason et al., 2008)
*λ*_*M*_	1.65		-
*λ*_*0*_	0.65		-
*K*_*act*_	0.10	day^−1^	(Baek et al., 2007)
*S*_*basal*_	0.862	N/m	-
*α*_*h*_	47.6	degree	-
$c_{1}^{e}$	657.9	J/kg	-
$c_{2}^{c}$	3090	J/kg	-
$c_{3}^{c}$	18.9		-
$c_{1}^{m}$	103	J/kg	-
$c_{2}^{m}$	11.5	J/kg	-
$c_{3}^{m}$	0.794		-

## Data Availability

The data that support the findings of this study are available from the corresponding author upon reasonable request.
