# Supplementary material for: A Biochemomechanical Model of Collagen Turnover in Arterial Adaptations to Hemodynamic Loading
Source: Res Sq. 2023 Feb 6:rs.3.rs-2535591. Preprint. [Version 1] doi: 10.21203/rs.3.rs-2535591/v1 (PMC9934758; doi:10.21203/rs.3.rs-2535591/v1)
Supplement: 1 [file NIHPPRS2535591V1-supplement-1.pdf]

Supplementary Figure

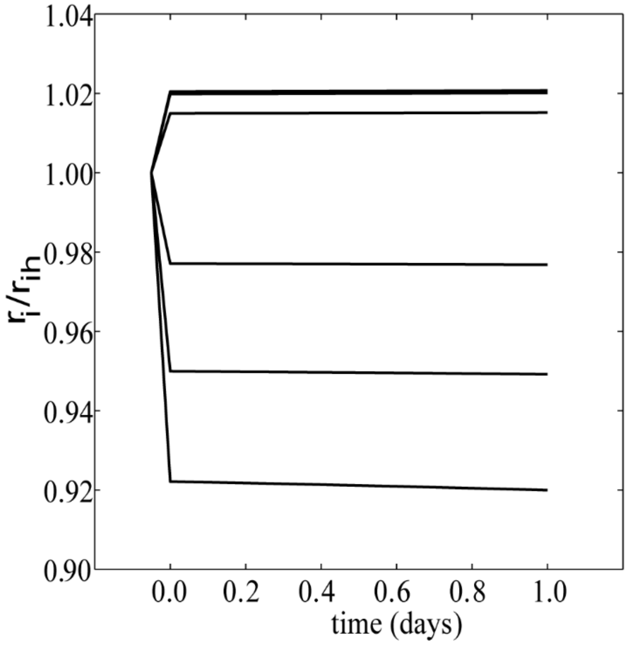

FIGURE S1. Variation in internal radius for one day as a function of a change in volumetric flow rate (from top to bottom 30%, 20%, and 10% increased volume flow rate, 10%, 20% and 30% decreased volume flow rate), all with $\boldsymbol{K_{\mu 1}^{k} = K_{\mu 2}^{k} = 0.01}$, $\boldsymbol{k_2^m = 13.6}$, $\boldsymbol{K_\sigma^c = K_\sigma^m = 1}$, $\boldsymbol{K_{\tau_w}^c = K_{\tau_w}^m = 3k_2^m}$, $\mu_1 = \boldsymbol{0.1}, \boldsymbol{\beta_2 = 0.1}$.
